# Clinical value of detecting IQGAP3, B7-H4 and cyclooxygenase-2 in the diagnosis and prognostic evaluation of colorectal cancer

**DOI:** 10.1186/s12935-019-0881-3

**Published:** 2019-06-14

**Authors:** Huihua Cao, Qing Wang, Zhenyan Gao, Xiang Xu, Qicheng Lu, Yugang Wu

**Affiliations:** Department of General Surgery, The Third Affiliated Hospital of Soochow University and The First People’s Hospital of Changzhou, 185 Juqian Street, Changzhou, 213000 Jiangsu China

**Keywords:** Colorectal cancer, IQ-motif-containing GTPase-activating protein, IQGAP3, B7-H4, Cyclooxygenase-2, Diagnostic markers

## Abstract

**Background:**

The IQ-motif-containing GTPase-activating protein (IQGAP) family comprises three members, IQGAP1, IQGAP2 and IQGAP3. IQGAP3 is the latest addition to the family. This study mainly investigated the novel marker IQGAP3 at serum and tumor tissue levels compared with the markers B7-H4 and cyclooxygenase-2 (COX-2) in patients with colorectal cancer (CRC) and in healthy individuals, aiming to evaluate the diagnostic and prognostic value of IQGAP3 for CRC.

**Materials and methods:**

Serum samples were collected prior to any therapy in 118 CRC patients and as part of a routine examination in 85 healthy individuals. Serum IQGAP3, B7-H4 and COX-2 levels were measured using commercially available ELISA kits. Immunohistochemistry was performed to detect the IQGAP3, B7-H4 and COX-2 in tumor tissues and normal para-carcinoma tissues. The receiver operating characteristics (ROC) curve and the area under the curve (AUC) were used to evaluate and compare the diagnostic value of different serum tumor markers. Univariate and multivariate analyses were performed to identify the prognostic risk factors for CRC.

**Results:**

IQGAP3, B7-H4 and COX-2 showed low or high expression in tumor tissues while no expression in normal para-carcinoma tissues. Serum levels of IQGAP3 in CRC group were significantly higher than those in healthy control group (*P* < 0.001). The IQGAP3 AUC was 0.799, while the B7-H4 AUC was 0.795 and the COX-2 AUC was 0.796. IQGAP3 seemed to be superior to B7-H4 and COX-2 in detecting CRC, with the highest sensitivity among the three markers. Multivariate analysis showed that T stage, N stage, differentiation degree, TNM stage and both serum and tissue IQGAP3, B7-H4 and COX-2 levels were significant prognostic factors for CRC.

**Conclusions:**

IQGAP3 has a better diagnostic efficacy than B7-H4 and COX-2 in detecting CRC and it has value in predicting the prognosis of patients with CRC.

**Electronic supplementary material:**

The online version of this article (10.1186/s12935-019-0881-3) contains supplementary material, which is available to authorized users.

## Background

Colorectal cancer (CRC) is the third most common cancer and the fourth leading cause of cancer-related mortality, with 30,000 new cases and 13,000 deaths each year [1, 2]. It is believed that regular and timely screening could avoid the occurrence or death of CRC, which is particularly necessary since early CRC is asymptomatic [3]. Although early diagnosis by detecting and advances in therapeutic strategies decreased mortality of CRC, the survival rate of CRC is poor yet [4, 5].

The most effective therapeutic strategy for CRC is surgical resection, but tumors at advanced stages may be inoperable [6]. The high mortality rate is usually resulted from late detection. Traditional detection methods such as endoscopic biopsy, magnetic resonance imaging and computed tomography are efficient in detecting tumor location and metastasis [3]. Besides, endoscopic biopsy and histopathological examination are considered to be the gold standard for diagnosing CRC [7]. However, the stress and pain caused by invasive methods along with high costs make it difficult to become a routine method for screening on a large population basis, especially for those asymptomatic individuals. Detecting serum tumor markers has become an easy non-invasive and common method for screening tumors [8]. Although carcinoembryonic antigen (CEA) is currently the most widely used tumor marker for gastrointestinal cancers [9, 10], it is not an appropriate index for the evaluation of CRC, especially in the early stages [11].

The IQ-motif-containing GTPase-activating protein (IQGAP) family is well conserved from yeast to humans, which comprises three members, IQGAP1, IQGAP2 and IQGAP3 [12]. IQGAP3 is the latest addition to the family, which is involved in the proliferation of epithelial cells [13–15]. In contrast to IQGAP2, IQGAP3 was found to be higher expressed in colorectal adenocarcinoma [16]. However, the role of IQGAP3 in tumorigenesis and its diagnostic and prognostic value of CRC remain to be determined. In addition to IQGAP3, a host of tumor markers have been detected and used in clinical practice [17]. B7-H4 is a co-stimulatory molecule of B7 family and the over-expression of B7-H4 is of prognostic significance in CRC patients [18]. Cyclooxygenase (COX) is a rate-limiting enzyme in the prostaglandin metabolism including COX-1 and COX-2. COX-2 is extensively studied which is up-regulated in response to growth factors, cytokines and tumor-promoting factors [19]. COX-2 over-expression is also associated with poor prognosis of CRC and may be used as an indicator for the diagnosis of CRC [20].

In this study, we mainly investigated the novel marker IQGAP3 at serum and tumor tissue levels compared with the markers B7-H4 and COX-2 in patients with CRC and in healthy individuals, aiming to evaluate the diagnostic and prognostic value of IQGAP3 for CRC.

## Materials and methods

### Patients

We retrospectively examined the medical records of patients with CRC at The Third Affiliated Hospital of Soochow University from May 2011 to May 2013. In order to obtain normal para-carcinoma tissues, same source of CRC patients from January 2018 to January 2019 were selected. The enrolled patients met the following criteria: (1) histological confirmed adenocarcinoma, (2) not received neoadjuvant chemoradiation, (3) underwent curative R0 resection, (4) with no evidence of distant metastasis. Patients were excluded if they had died or had incomplete clinicopathological data. Tumor stage was classified according to the 8th edition of TNM staging system. Follow-up was conducted by telephone calls, e-mails, and on-site visits. Besides, eighty-five healthy age-and sex-matched individuals were recruited from people who came for general health examinations in our hospital, with an exclusion of history of past diseases. Informed written consent was obtained from all patients and healthy individuals and this study was approved by the Ethics Committee of The Third Affiliated Hospital of Soochow University. The approval number was 201165.

### Immunohistochemistry staining

Tumor tissue samples and normal para-carcinoma tissues were collected after surgery in CRC patients. Immunohistochemistry was performed to detect the IQGAP3, B7-H4 and COX-2 in tumor tissues from patients of May 2011 to May 2013 and normal para-carcinoma tissues from patients of January 2018 to January 2019. Specimens were cut into 5 mm thick slices and then subjected to routine deparaffinization and rehydration. The original anti-IQGAP3 antibody (ab88353, Abcam, Shanghai), anti-B7-H4 antibody (EPR20236, Abcam, Shanghai) and anti-COX-2 antibody (ab15191, Abcam, Shanghai) were used in a 1:200 dilution. After incubation at 4 °C, a microscopic examination was performed to determine the percentage of cells positively stained for IQGAP3, B7-H4 and COX-2.

All slices were analyzed by two clinical pathologists independently. When disagreement occurred, the third pathologist was asked to confirm the assessment. The inter-assessor agreement rate between the two pathologists was 95.6%. As for the classification of cases into low or high expression group, we referred to the study reported by Albasri AM [19]. The extent of staining was scored as follows: 0 (no staining), 1 (1–10% positive staining), 2 (10–50% positive staining), 3 (50–70% positive staining) and 4 (70–100% positive staining). The intensity of staining was scored as follows: 0 (negative), 1 (weakly positive), 2 (moderately positive) and 3 (strongly positive). The final expression score was determined as follows: − (scores 0), + (scores 1–3), ++ (scores 4–6) and +++ (scores > 6). For statistical analysis, the cases that scored as − and + were combined as the low expression group, which was compared to the high expression group consisted of the cases scored as ++ and +++.

### Detection of serum tumor markers

Serum samples were collected prior to any therapy in CRC patients and as part of a routine examination in healthy individuals. All samples were collected in anti-coagulated tubes containing ethylene diamine tetraacetie acid (EDTA). Serum IQGAP3, B7-H4 and COX-2 levels were measured using commercially available ELISA kits (RENJIEBIO, Shanghai, China) at the tumor laboratory of The Third Affiliated Hospital of Soochow University. Coefficient of variation (CV) was used to validate the precision of the ELISA assays, which was defined as the ration of standard deviation and mean value. Serum CEA and CA19-9 levels were achieved directly for they were conventional examination items in our hospital.

### Determination of the cut-off value

Youden Index, which was calculated as sensitivity + specificity − 1, was used to determine the optimum cut-off values of the three tumor markers. The maximum Youden Index corresponds to the optimum cut-off value. We divided the patients into low expression group and high expression group based on the cut-off values of serum tumor markers.

### Statistical analysis

All analyses were carried out using SPSS version 17.0 software (IBM SPSS, Chicago, IL). The data of tumor markers were summarized and expressed in the form of mean ± SD. Mann–Whitney U test or Kruskal–Wallis test was used to compare the relationship between the serum tumor markers levels and clinicopathological parameters. Chi squared test and two-sided Fisher’s exact test were used to evaluate the association between staining intensity and clinicopathological parameters. The correlation between IQGAP3, B7-H4 and COX-2 was respectively evaluated by using Spearman correlation test. The receiver operating characteristics (ROC) curve and the area under the curve (AUC) were used to evaluate and compare the diagnostic value of different serum tumor markers. Kaplan–Meier analysis followed by a log-rank test was used to compare the patient survival between different groups. Univariate and multivariate analyses were performed by using a Cox proportional hazards model. All statistical analyses were two-sided and values of *P* < 0.05 were considered statistically significant.

## Results

### Patient characteristics

One hundred eighteen CRC patients from May 2011 to May 2013 and sixty-two CRC patients from January 2018 to January 2019 who met the inclusion criteria shown above and eighty-five healthy individuals were analyzed in this study. Clinicopathological characteristics of 118 patients and their correlation with serum tumor marker levels were listed in Table 1. There were 49 female patients and 69 male patients, with age ≤ 60 and > 60 patients accounting for 44.9% and 55.1%, respectively. Owing to the late diagnosis, the majority of patients were in a later stage, with stage T3 and T4 patients accounting for 28.8% and 25.4%, respectively. The percentage of patients in stage I–II and III were 40.7% versus 59.3%, respectively. There appears to be an overrepresentation of the later stage CRC cases which would inflate the sensitivity and specificity of the discussed biomarkers.

**Table 1:** Correlation of serum IQGAP3, B7-H4 and COX-2 levels with clinicopathological features in 118 CRC patients

Parameters	Patients	IQGAP3	*P*	B7-H4	*P*	COX-2	*P*
	n (%)	Mean ± SD, pg/ml		Mean ± SD, ng/ml		Mean ± SD, ng/ml	
Age (years)			0.023^a^		0.282^a^		0.102^a^
≤ 60	53 (44.9)	300.26 ± 104.31		99.34 ± 14.23		47.12 ± 11.23	
> 60	65 (55.1)	322.47 ± 99.67		97.25 ± 15.88		48.38 ± 11.35	
Sex			0.506^a^		0.173^a^		0.188^a^
Female	49 (41.5)	316.03 ± 104.98		96.53 ± 13.44		46.99 ± 12.56	
Male	69 (58.5)	311.54 ± 101.42		98.96 ± 15.43		48.32 ± 13.45	
Tumor site			0.208^a^		0.196^a^		0.234^a^
Colon	66 (55.9)	316.38 ± 99.03		97.24 ± 15.36		47.34 ± 12.03	
Rectum	52 (44.1)	312.35 ± 103.54		95.87 ± 12.14		48.87 ± 13.67	
Tumor size (cm)			0.052^a^		0.358^a^		0.162^a^
≤ 4	63 (53.4)	311.93 ± 90.17		98.76 ± 15.67		47.33 ± 12.28	
> 4	55 (46.6)	325.00 ± 87.65		101.11 ± 10.42		50.12 ± 14.66	
T stage			0.001^b^		0.024^b^		0.016^b^
T1	22 (18.6)	180.33 ± 71.10		81.23 ± 9.32		37.12 ± 11.02	
T2	32 (27.1)	185.57 ± 84.10		89.44 ± 10.53		45.33 ± 9.89	
T3	34 (28.8)	272.78 ± 79.20		96.56 ± 17.53		51.02 ± 10.17	
T4	30 (25.4)	382.71 ± 76.76		103.30 ± 11.88		53.44 ± 8.76	
N stage			< 0.001^b^		0.004^b^		0.008^b^
N0	48 (40.7)	252.97 ± 88.16		87.54 ± 12.23		38.41 ± 9.76	
N1	39 (33.1)	323.11 ± 80.03		97.65 ± 14.08		45.67 ± 11.65	
N2	31 (26.3)	392.02 ± 88.68		104.45 ± 9.88		51.87 ± 10.03	
Differentiation degree			0.086^b^		0.052^b^		0.109^b^
Well	56 (47.5)	272.75 ± 75.60		93.33 ± 13.33		40.89 ± 10.33	
Moderate	50 (42.4)	351.91 ± 90.22		93.37 ± 12.23		45.38 ± 11.65	
Poor	12 (10.2)	358.57 ± 91.57		100.80 ± 13.89		51.70 ± 11.78	
Retrieved LN			0.017^a^		0.085^a^		0.256^a^
≤ 12	61 (51.7)	354.78 ± 88.75		97.56 ± 12.80		47.91 ± 12.36	
> 12	57 (48.3)	266.63 ± 96.23		96.38 ± 16.92		48.56 ± 11.67	
TNM stage			< 0.001^a^		0.002^a^		< 0.001^a^
I + II	48 (40.7)	270.22 ± 102.70		88.23 ± 15.67		39.21 ± 14.22	
III	70 (59.3)	338.86 ± 92.78		108.12 ± 14.21		51.87 ± 11.78	

One hundred eighteen CRC patients who met the inclusion criteria were analyzed in this study. The correlation between clinicopathological characteristics and serum tumor markers were analyzed

LN, lymph nodes; TNM, tumor-node-metastasis; SD, standard deviation

^a^By Mann–Whitney U test

^b^By Kruskal–Wallis test

### Levels of tissue tumor markers in different groups

**Fig. 1:**
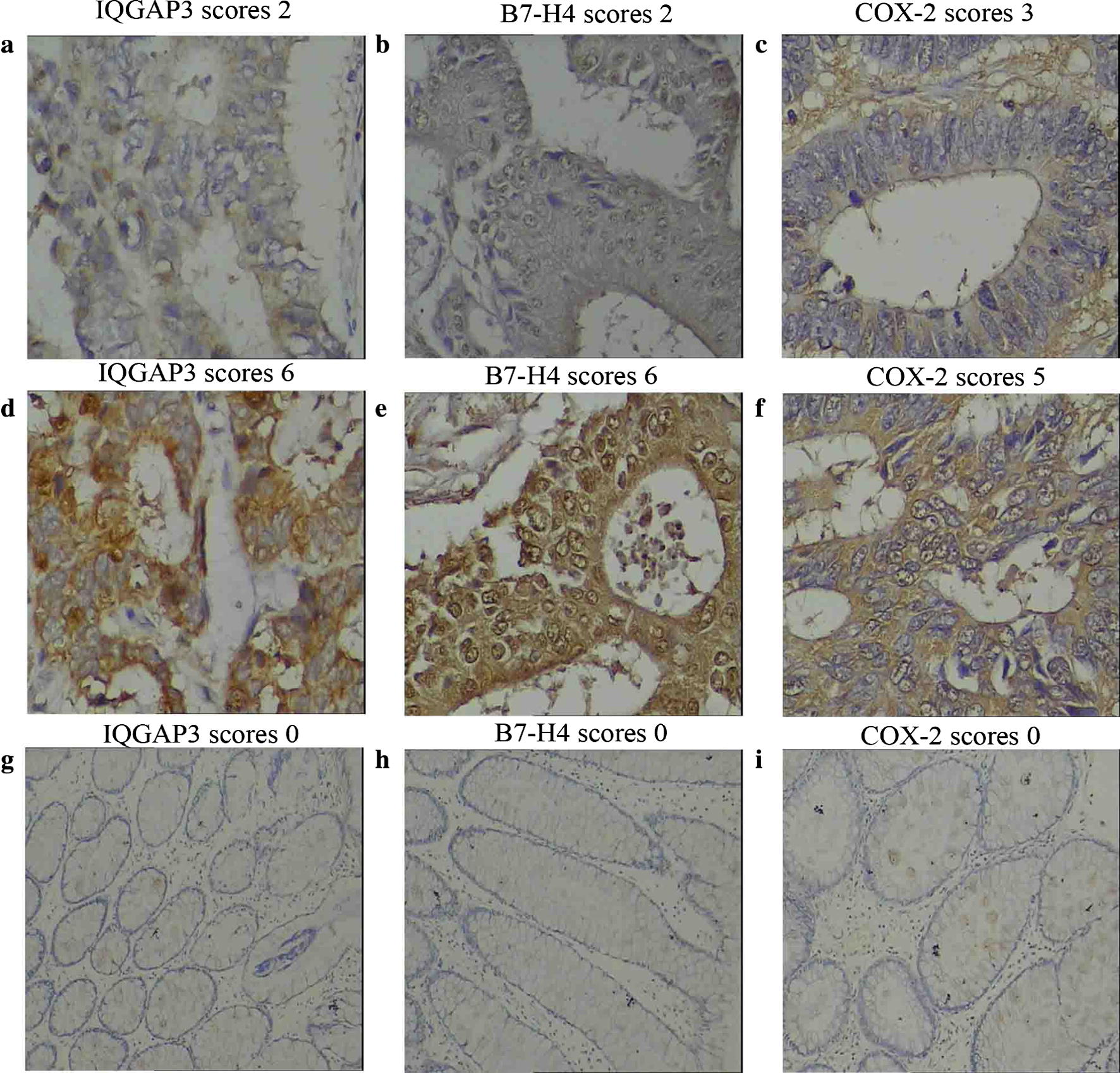
Levels of tissue tumor markers in different group. 118 CRC patients from May 2011 to May 2013 and 62 CRC patients from January 2018 to January 2019 were analyzed. Specimens were subjected to routine deparaffinization and rehydration. The original anti-IQGAP3 antibody, anti-B7-H4 antibody and anti-COX-2 antibody were used in a 1:200 dilution. Immunohistochemical expression of IQGAP3, B7-H4 and COX-2 in tumor tissues and normal para-carcinoma tissues was observed in the cytoplasm with different intensities. Positive staining showed a brown color. Low expression of immunohistochemical staining of three molecules in **a** IQGAP3, **b** B7-H4, **c** COX-2, respectively (×400), with expression quantity of 20%, 10%, 10%, respectively. High expression of immunohistochemical staining of three molecules in **d** IQGAP3, **e** B7-H4, **f** COX-2, respectively (×400), with expression quantity of 70%, 80%, 80%, respectively. Negative immunohistochemical staining of normal para-carcinoma tissues in **g** IQGAP3, **h** B7-H4, and **i** COX-2, respectively (×100), with expression quantity of zero in all

IQGAP3, B7-H4 and COX-2 immunohistochemistry staining was quantitatively assessed and divided into low and high groups (Table 2). Positive staining showed a brown color. Immunohistochemical expression of IQGAP3 in tumor tissues was observed in the cytoplasm with different intensities, with 61 cases showing low expression (20% IQGAP3, Fig. 1a) and 57 cases showing high expression (70% IQGAP3, Fig. 1d). However, no IQGAP3 expression was found in normal para-carcinoma tissues (Fig. 1g). Similar condition was observed in B7-H4 immunohistochemistry staining in the cytoplasm, 52 cases showed low B7-H4 expression (10% B7-H4, Fig. 1b) and 66 cases showed high B7-H4 expression (80% B7-H4, Fig. 1e). Normal para-carcinoma tissues showed no B7-H4 expression (Fig. 1h). COX-2 immunoreactivity was also observed in the cytoplasm in all CRC cases, with 55 cases low expression (10% COX-2, Fig. 1c) and 63 cases high expression (80% COX-2, Fig. 1f). In comparison, COX-2 staining of normal para-carcinoma tissues was negative (Fig. 1i).

**Table 2:** Correlation of tissue IQGAP3, B7-H4 and COX-2 expression levels with clinicopathological features in 118 CRC patients

Parameters	Patients	IQGAP3		*P*	B7-H4		*P*	COX-2		*P*
	n (%)	Low	High		Low	High		Low	High	
	118	61	57		52	66		55	63	
Age (years)				0.605			0.083			0.394
≤ 60	53	26	27		28	25		27	26	
> 60	65	35	30		24	41		28	37	
Sex				0.081			0.597			0.418
Female	49	30	19		23	26		25	24	
Male	69	31	38		29	40		30	39	
Tumor site				0.678			0.685			0.777
Colon	66	33	33		28	38		30	36	
Rectum	52	28	24		24	28		25	27	
Tumor size (cm)				0.369			0.115			0.213
≤ 4	63	35	28		32	31		26	37	
> 4	55	26	29		20	35		29	26	
T stage				< 0.001			< 0.001			< 0.001
T1	22	18	4		20	2		18	4	
T2	32	26	6		18	14		25	7	
T3	34	15	19		11	23		9	25	
T4	30	2	28		3	27		3	27	
N stage				0.003			< 0.001			< 0.001
N0	48	30	18		33	15		36	12	
N1	39	23	16		15	24		14	25	
N2	31	8	23		4	27		5	26	
Differentiation degree				0.427			0.102			0.239
Well	56	30	26		30	26		29	27	
Moderate	50	26	24		19	31		23	27	
Poor	12	5	7		3	9		3	9	
Retrieved LN				0.201			0.678			0.873
≤ 12	61	35	26		28	33		28	33	
> 12	57	26	31		24	33		27	30	
TNM stage				0.007			0.001			< 0.001
I + II	48	32	16		30	18		39	9	
III	70	29	41		22	48		16	54	

One hundred eighteen CRC patients who met the inclusion criteria were analyzed in this study. The correlation between clinicopathological characteristics and tissue tumor markers were analyzed

LN, lymph nodes; TNM, tumor-node-metastasis

### Levels of serum tumor markers in different groups

As shown in Table 3, 118 CRC patients were set as CRC group including T1–T4 groups and 85 healthy individuals were set as control group. Serum levels of IQGAP3, B7-H4, COX-2, CEA and CA19-9 in CRC group were significantly higher than those in control group (IQGAP3: 312.68 ± 101.91 vs. 191.97 ± 103.96, *P* < 0.001; B7-H4: 97.85 ± 15.56 vs. 64.78 ± 16.73, *P* = 0.003; COX-2: 48.55 ± 13.43 vs. 25.78 ± 16.09, *P* < 0.001; CEA: 6.62 ± 4.49 vs. 3.41 ± 1.27, *P* < 0.001; CA19-9: 29.92 ± 8.97 vs. 19.24 ± 10.08, *P* < 0.001). However, we can see that IQGAP3 in T1 and T2 groups were not higher than that in control group (T1 180.33 ± 71.10: Control 191.97 ± 103.96, *P* = 0.284; T2 185.57 ± 84.10: Control 191.97 ± 103.96, *P* = 0.412). Besides, CVs of each group were also shown.

**Table 3:** IQGAP3, B7-H4, COX-2, CEA and CA19-9 levels in different patient groups

Tumor markers		Control group	CRC group				
			All	T1	T2	T3	T4
	Cases (n)	85	118	22	32	34	30
IQGAP3	Levels (mean ± SD, pg/ml)	191.97 ± 103.96	312.68 ± 101.91	180.33 ± 71.10	185.57 ± 84.10	272.78 ± 79.20	382.71 ± 76.76
	P		< 0.001^#^	= 0.284*	= 0.412**	< 0.001***	< 0.001^##^
	CV	54.2	32.6	39.4	45.3	29.0	20.1
B7-H4	Levels (mean ± SD, ng/ml)	64.78 ± 16.73	97.85 ± 15.56	81.23 ± 9.32	89.44 ± 10.53	96.56 ± 17.53	103.30 ± 11.88
	P		< 0.001^#^	= 0.003*	= 0.001**	< 0.001***	< 0.001^##^
	CV	25.8	15.9	11.5	11.8	18.2	11.5
COX-2	Levels (mean ± SD, ng/ml)	25.78 ± 16.09	48.55 ± 13.43	37.12 ± 11.02	45.33 ± 9.89	51.02 ± 10.17	53.44 ± 8.76
	P		< 0.001^#^	= 0.001*	< 0.001**	< 0.001***	< 0.001^##^
	CV	62.4	27.7	29.7	21.8	19.9	16.4
CEA	Levels (mean ± SD, ng/ml)	3.41 ± 1.27	6.22 ± 4.49	5.34 ± 1.17	5.92 ± 1.85	6.08 ± 3.82	6.34 ± 2.14
	P		< 0.001^#^	= 0.004*	= 0.001**	< 0.001***	< 0.001^##^
	CV	37.2	72.2	21.9	31.3	62.8	33.8
CA19-9	Levels (mean ± SD, U/ml)	19.24 ± 10.08	29.92 ± 8.97	27.19 ± 8.87	29.81 ± 9.43	30.90 ± 10.16	31.5 ± 9.58
	P		< 0.001^#^	< 0.001*	< 0.001**	< 0.001***	< 0.001^##^
	CV	52.4	30.0	32.6	31.6	32.9	30.4

118 CRC patients were set as CRC group including T1–T4 groups and 85 healthy individuals were set as control group. Serum levels of IQGAP3, B7-H4, COX-2, CEA and CA19-9 in CRC group were significantly higher than those in control group (IQGAP3: 312.68 ± 101.91 vs. 191.97 ± 103.96 *P* < 0.001; B7-H4: 97.85 ± 15.56 vs. 64.78 ± 16.73, *P* = 0.003; COX-2: 48.55 ± 13.43 vs. 25.78 ± 16.09 *P* < 0.001; CEA: 6.62 ± 4.49 vs. 3.41 ± 1.27, *P* < 0.001; CA19-9: 29.92 ± 8.97 vs. 19.24 ± 10.08, *P* < 0.001). However, IQGAP3 in T1 and T2 groups were not higher than that in control group (T1 180.33 ± 71.10: Control 191.97 ± 103.96, *P* = 0.284; T2 185.57 ± 84.10: Control 191.97 ± 103.96, *P* = 0.412). CVs of each group were also shown

CRC, colorectal cancer; SD, standard deviation; CV, coefficient of variation

* Control group vs. T1 group

** Control group vs. T2 group

*** Control group vs. T3 group

^#^Control group vs. all group

^##^Control group vs. T4 group

### Correlations between serum tumor markers

**Fig. 2:**
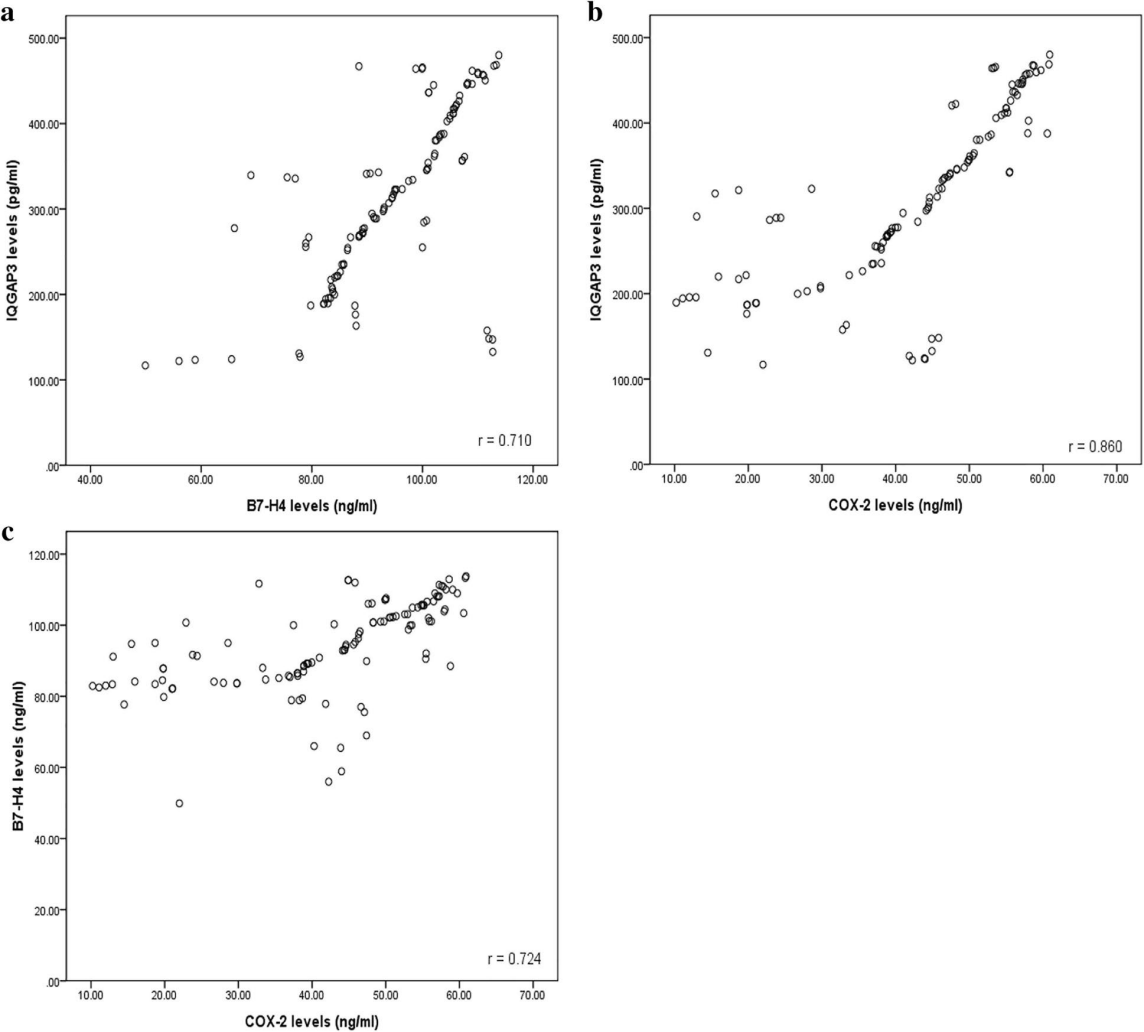
Correlations between serum tumor markers. 118 CRC patients from May 2011 to May 2013 were analyzed. **a** There was a correlation between IQGAP3 and B7-H4 levels (r = 0.710, *P* < 0.001). Points show IQGAP3 or B7-H4 levels of each patient, with X axis indicating B7-H4 levels (ng/ml) and Y axis indicating IQGAP3 levels (pg/ml). **b** There was a correlation between IQGAP3 and COX-2 levels (r = 0.860, *P* < 0.001). Points show IQGAP3 or COX-2 levels of each patient, with X axis indicating COX-2 levels (ng/ml) and Y axis indicating IQGAP3 levels (pg/ml). **c** There was a correlation between B7-H4 and COX-2 levels (r = 0.724, *P* < 0.001). Points show B7-H4 or COX-2 levels of each patient, with X axis indicating COX-2 levels (ng/ml) and Y axis indicating B7-H4 levels (ng/ml)

The results of Spearman correlation analysis were shown in Fig. 2. IQGAP3, B7-H4 and COX-2 these three tumor markers were compared in three groups. Figure 2a indicated that there was a correlation between IQGAP3 and B7-H4 levels (r = 0.710, *P* < 0.001). Points show IQGAP3 or B7-H4 levels of each patient, with X axis indicating B7-H4 levels (ng/ml) and Y axis indicating IQGAP3 levels (pg/ml). Figure 2b showed that there was a correlation between IQGAP3 and COX-2 levels (r = 0.860, *P* < 0.001). Points show IQGAP3 or COX-2 levels of each patient, with X axis indicating COX-2 levels (ng/ml) and Y axis indicating IQGAP3 levels (pg/ml). Figure 2c also showed that there was a correlation between B7-H4 and COX-2 levels (r = 0.724, *P* < 0.001). Points show B7-H4 or COX-2 levels of each patient, with X axis indicating COX-2 levels (ng/ml) and Y axis indicating B7-H4 levels (ng/ml).

### Clinicopathological significance of different tumor markers expression

The correlation of serum IQGAP3, B7-H4 and COX-2 expression levels with clinicopathological features were shown in Table 1. Serum IQGAP3, B7-H4 and COX-2 levels were correlated with T stage, N stage, and TNM stage (*P* < 0.05). The correlation of serum IQGAP3, CEA and CA19-9 expression levels with clinicopathological features were shown in Additional file 1. Serum CEA and CA19-9 levels were correlated with T stage, N stage, differentiation degree and TNM stage (*P* < 0.05). The correlation of tissue IQGAP3, B7-H4 and COX-2 expression levels with clinicopathological features were shown in Table 2. Tissue IQGAP3, B7-H4 and COX-2 expression levels were also correlated with T stage, N stage and TNM stage (*P* < 0.05).

### ROC curves of single tumor markers in CRC patients

**Fig. 3:**
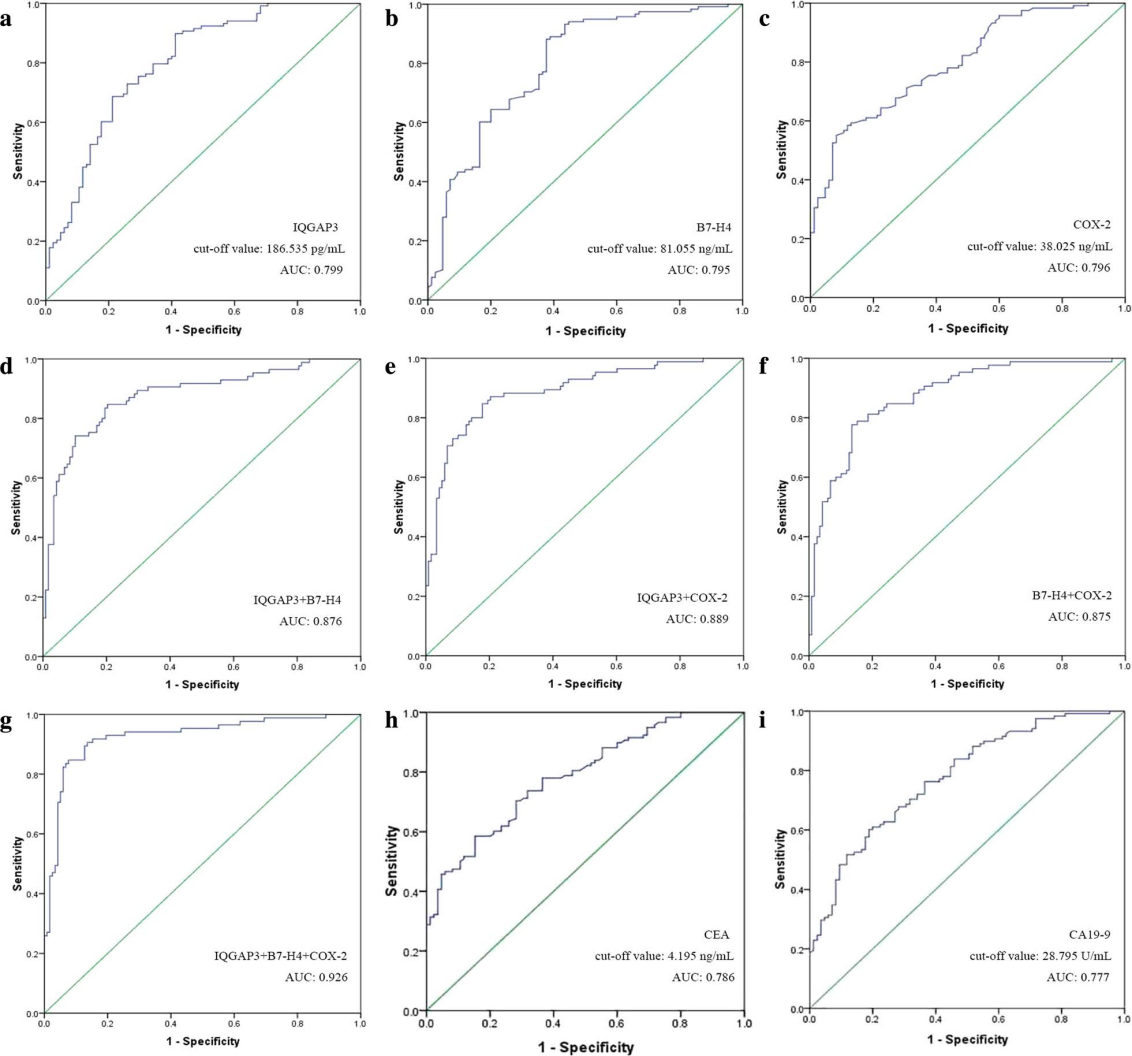
ROC curves of tumor markers in CRC patients. 118 CRC patients from May 2011 to May 2013 were analyzed. **a** The IQGAP3 AUC was 0.799, with 95% Confidence interval (CI) 0.736–0.861 (*P* < 0.001). **b** The B7-H4 AUC was 0.795 (95% CI 0.731–0.858, *P* < 0.001). **c** The COX-2 AUC was 0.796 (95% CI 0.737–0.856, *P* < 0.001). **d** The IQGAP3 + B7-H4 AUC was 0.876 (95% CI 0.825–0.927, *P* < 0.001). **e** The IQGAP3 + COX-2 AUC was 0.889 (95% CI 0.842–0.936, *P* < 0.001). **f** The B7-H4 + COX-2 AUC was 0.875 (95% CI 0.827–0.924, *P* < 0.001). **g** The AUC of IQGAP3 + B7-H4 + COX-2 was 0.926 (95% CI 0.887–0.966, *P* < 0.001). **h** The CEA AUC was 0.786, (95% CI 0.725–0.847, *P* < 0.001). **i** The CA19-9 AUC was 0.777, (95% CI 0.714–0.840, *P* < 0.001)

We analyzed the ROC curves of serum IQGAP3, B7-H4, COX-2, CEA and CA19-9 in CRC patients (Table 4). The IQGAP3 AUC was 0.799, with 95% confidence interval (CI) 0.736–0.861 (*P* < 0.001) (Fig. 3a). The B7-H4 AUC was 0.795 (95% CI 0.731–0.858, *P* < 0.001) (Fig. 3b) and the COX-2 AUC was 0.796 (95% CI 0.737–0.856, *P* < 0.001) (Fig. 3c). The CEA AUC was 0.786 (95% CI 0.725–0.847, *P* < 0.001) (Fig. 3h). The CA19-9 AUC was 0.777 (95% CI 0.714–0.840, *P* < 0.001) (Fig. 3i).

**Table 4:** Efficiency of serum IQGAP3, B7-H4, COX-2, CEA and CA19-9 in the diagnosis of CRC

Factor	Sensitivity (%)	Specificity (%)	Youden Index	Accuracy (%)
IQGAP3	89.8	58.8	0.486	0.799
B7-H4	88.1	62.4	0.505	0.795
COX-2	79.2	69.4	0.486	0.796
IQGAP3 + B7-H4	92.9	62.5	0.554	0.876
IQGAP3 + COX-2	91.8	56.8	0.486	0.889
B7-H4 + COX-2	90.6	63.5	0.541	0.875
IQGAP3 + B7-H4 + COX-2	94.1	74.5	0.686	0.926
CEA	60.3	71.8	0.321	0.786
CA19-9	50.2	81.2	0.314	0.777

We analyzed the ROC curves of serum IQGAP3, B7-H4, COX-2, CEA and CA19-9 in CRC patients. Sensitivity, specificity, Youden Index and the AUC value

CRC, colorectal cancer; CI, confidence interval

When the cut-off value for IQGAP3 was chosen as 186.535 pg/ml, as determined by the maximum value of Youden index, the sensitivity and specificity for IQGAP3 were 89.8 and 58.8%, respectively. When the cut-off value for B7-H4 was selected at 81.055 ng/ml, the sensitivity and specificity for B7-H4 were 88.1 and 62.4%, respectively. When the cut-off value for COX-2 was selected at 38.025 ng/ml, the sensitivity and specificity were 79.2 and 69.4%, respectively. When the cut-off value for CEA was chosen as 4.195 ng/ml, the sensitivity and specificity for CEA were 60.3 and 71.8%, respectively. When the cut-off value for CA19-9 was selected at 28.795 U/ml, the sensitivity and specificity for CA19-9 were 50.2 and 81.2%, respectively. IQGAP3 seemed to be inferior to B7-H4, COX-2, CEA and CA19-9 in specificity when detecting CRC, however, its sensitivity and AUC were all the highest among the three markers.

### ROC curves of two-combined tumor markers in CRC patients

We analyzed the ROC curves of serum IQGAP3 + B7-H4, IQGAP3 + COX-2, B7-H4 + COX-2 in CRC patients (Table 4). As shown in Fig. 3d, the IQGAP3 + B7-H4 AUC was 0.876 (95% CI 0.825–0.927, *P* < 0.001). The IQGAP3 + COX-2 AUC was 0.889 (95% CI 0.842–0.936, *P* < 0.001) (Fig. 3e) and the B7-H4 + COX-2 AUC was 0.875 (95% CI 0.827–0.924, *P* < 0.001) (Fig. 3f). When determined by the maximum value of Youden index, the sensitivity and specificity for IQGAP3 + B7-H4 were 92.9 and 62.5%, respectively; the sensitivity and specificity for IQGAP3 + COX-2 were 91.8 and 56.8%, respectively; the sensitivity and specificity for B7-H4 + COX-2 were 90.6 and 63.5%, respectively. Thus, IQGAP3 + COX-2 showed superiority in detecting CRC.

### ROC curves of three-combined tumor marker in CRC patients

We analyzed the ROC curves of IQGAP3 + B7-H4 + COX-2 in CRC patients (Table 4). As shown in Fig. 3g, the AUC of IQGAP3 + B7-H4 + COX-2 was 0.926 (95% CI 0.887–0.966, *P* < 0.001). When determined by the maximum value of Youden index, the sensitivity and specificity for IQGAP3 + B7-H4 + COX-2 were 94.1 and 74.5%. IQGAP3 + B7-H4 + COX-2 was superior to other markers in detecting CRC.

### Survival analysis

**Fig. 4:**
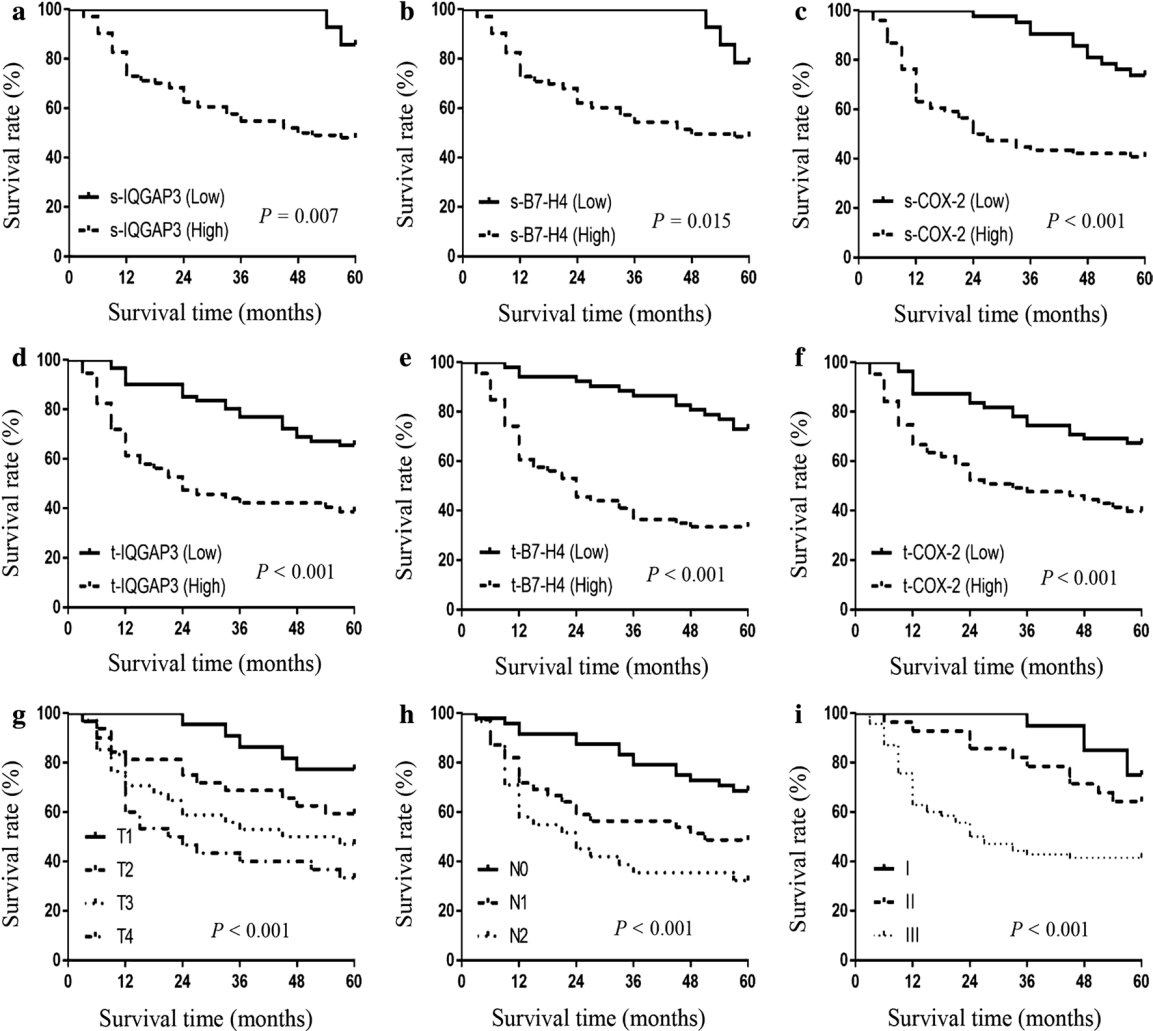
Overall survival rates for patients in different groups. 118 CRC patients from May 2011 to May 2013 were analyzed. We divided patients into several subgroups according to tumor marker levels, T stage, N stage and TNM stage. **a** The 5-year survival rates in s-IQGAP3 low group were significantly higher than those in s-IQGAP3 high group (s-IQGAP3 low 85.7% vs. s-IQGAP3 high 48.1%, *P* = 0.007). **b** The 5-year survival rates in s-B7-H4 low group were significantly higher than those in s-B7-H4 high group (s-B7-H4 low 78.6% vs. s-B7-H4 high 49.0%, *P* = 0.015). **c** The 5-year survival rates in s-COX-2 low group were significantly higher than those in s-COX-2 high group (s-COX-2 low 73.8% vs. s-COX-2 high 40.8%, *P* < 0.001). **d** The 5-year survival rates in t-IQGAP3 low group were significantly higher than those in t-IQGAP3 high group (t-IQGAP3 low 65.6% vs. t-IQGAP3 high 38.6%, *P* < 0.001). **e** The 5-year survival rates in t-B7-H4 low group were significantly higher than those in t-B7-H4 high group (t-B7-H4 low 73.1% vs. t-B7-H4 high 36.4%, *P* < 0.001). **f** The 5-year survival rates in t-COX-2 low group were significantly higher than those in t-COX-2 high group (t-COX-2 low 67.3% vs. t-COX-2 high 39.7%, *P* < 0.001). **g** The 5-year survival rates in the four T stages were significantly different (T1 77.3% vs. T2 59.4% vs. T3 47.1% vs. T4 33.3%). **h** The 5-year survival rates in the three N stages were significantly different (N0 68.8% vs. N1 48.7% vs. N2 32.3%). **i** The 5-year survival rates in the three TNM stages were significantly different (stage I 80.0% vs. stage II 60.7% vs. stage III 41.4%)

All 118 patients from May 2011 to May 2013 in this study were followed up for more than 5 years. The 5-year overall survival (OS) rate was 52.5%. Univariate analysis showed that tumor size, T stage, N stage, differentiation degree, TNM stage, both serum and tissue IQGAP3, B7-H4 and COX-2 levels and serum CEA and CA19-9 levels were significant prognostic factors for CRC. However, based on multivariate analysis, tumor size was not statistically significant as a prognostic risk factor (Table 5). Our study showed that the 5-year OS rate in both s-IQGAP3 and t-IQGAP3 low groups were significantly higher than those in s-IQGAP3 and t-IQGAP3 high groups, respectively (s-IQGAP3 low 85.7% vs. s-IQGAP3 high 48.1%, *P* = 0.007, Fig. 4a; t-IQGAP3 low 65.6% vs. t-IQGAP3 high 38.6%, *P* < 0.001, Fig. 4d). Similarly, the 5-year survival rates between B7-H4 or COX-2 levels low and high groups also showed statistical significance, either in serum levels or tissue levels (s-B7-H4 low 78.6% vs. s-B7-H4 high 49.0%, *P* = 0.015, Fig. 4b; t-B7-H4 low 73.1% vs. t-B7-H4 high 36.4%, *P* < 0.001, Fig. 4e; s-COX-2 low 73.8% vs. s-COX-2 high 40.8%, *P* < 0.001, Fig. 4c; t-COX-2 low 67.3% vs. t-COX-2 high 39.7%, *P* < 0.001, Fig. 4f). We also compared the survival curves according to TNM stage in these patients. The 5-year survival rates in the four T stages were significantly different (T1 77.3% vs. T2 59.4% vs. T3 47.1% vs. T4 33.3%, *P* < 0.001, Fig. 4g). The 5-year survival rates in the three N stages were significantly different (N0 68.8% vs. N1 48.7% vs. N2 32.3%, *P* < 0.001, Fig. 4h). The 5-year survival rates in the three TNM stages were significantly different (stage I 80.0% vs. stage II 60.7% vs. stage III 41.4%, *P* < 0.001, Fig. 4i).

**Table 5:** Univariate and multivariate analyses of prognostic factors

Parameters	Patients	5-year OS rate (%)	Univariate analysis	Multivariate analysis		
	N (%)		*P*	HR	95% CI	*P*
Age (years)			0.560			
≤ 60	53 (44.9)	52.8				
> 60	65 (55.1)	52.3				
Sex			0.132			
Female	49 (41.5)	53.1				
Male	69 (58.5)	52.2				
Tumor site			0.102			
Colon	66 (55.9)	53.0				
Rectum	52 (44.1)	51.9				
Tumor size (cm)			0.032	1.752	0.892–2.321	0.087
≤ 4	63 (53.4)	57.1				
> 4	55 (46.6)	47.3				
T stage			< 0.001	2.337	1.273–3.755	0.001
T1	22 (18.6)	77.3				
T2	32 (27.1)	59.4				
T3	34 (28.8)	47.1				
T4	30 (25.4)	33.3				
N stage			< 0.001	1.988	1.034–2.433	< 0.001
N0	48 (40.7)	68.8				
N1	39 (33.1)	48.7				
N2	31 (26.3)	32.3				
Differentiation degree			0.002	1.786	1.296–2.280	0.035
Well	56 (47.5)	66.1				
Moderate	50 (42.4)	42.0				
Poor	12 (10.2)	33.3				
Retrieved LN			0.182			
≤ 12	61 (51.7)	52.5				
> 12	57 (48.3)	52.6				
TNM stage			< 0.001	2.271	1.360–3.365	< 0.001
I + II	48 (40.7)	68.8				
III	70 (59.3)	41.4				
s-IQGAP3 (pg/ml)			0.007	1.915	1.441–2.673	0.012
≤ 186	14 (11.9)	85.7				
> 186	104 (88.1)	48.1				
s-B7-H4 (ng/ml)			0.015	2.013	1.587–3.014	0.038
≤ 81	14 (11.9)	78.6				
> 81	104 (88.1)	49.0				
s-COX-2 (ng/ml)			< 0.001	1.810	1.299–2.836	< 0.001
≤ 38	42 (35.6)	73.8				
> 38	76 (64.4)	40.8				
t-IQGAP3			< 0.001	2.520	1.298–3.442	< 0.001
Low	61 (51.7)	65.6				
High	57 (48.3)	38.6				
t-B7-H4			< 0.001	2.112	1.083–3.960	< 0.001
Low	52 (44.1)	73.1				
High	66 (55.9)	36.4				
t-COX-2			< 0.001	1.886	1.155–2.876	< 0.001
Low	55 (46.6)	67.3				
High	63 (53.4)	39.7				
s-CEA (ng/ml)			< 0.001	2.232	1.187–4.552	< 0.001
≤ 4.195	34 (28.8)	67.6				
> 4.195	84 (71.2)	46.6				
s-CA19-9 (U/ml)			< 0.001	2.118	1.333–4.237	< 0.001
≤ 28.795	47 (39.8)	66.0				
> 28.795	71 (60.2)	43.7				

Univariate analysis showed that tumor size, T stage, N stage, differentiation degree, TNM stage, both serum and tissue IQGAP3, B7-H4 and COX-2 levels and serum CEA and CA19-9 levels were significant prognostic factors for CRC. However, based on multivariate analysis, tumor size was not statistically significant as a prognostic risk factor

LN, lymph nodes; TNM, tumor-node-metastasis; OS, overall survival

## Discussion

CRC is one of the most commonly diagnosed cancers in the world [21]. Although progress in diagnosis had been achieved in the past decades, 60% CRC patients were diagnosed with lymph nodes or distant metastasis [22]. Besides, there has been an increase in the morbidity and mortality in Asia owing to changes in diet and lifestyle [21]. Thus, effective and stable biomarkers may promote the diagnostic and predictive efficacy of CRC.

In fact, CEA is the most widely used diagnostic marker in CRC [23]. CA19-9 was also proved to be effective in monitoring CRC process [24]. However, CEA has a sensitivity of only 40% to 75% for CRC, although a specificity of 90% [25]. CA19-9 has a sensitivity and specificity of 23 and 96% for CRC [26]. Due to the low sensitivity, these tumor markers cannot be used for diagnostic purposes [24]. Thus, in this study we take IQGAP3, B7-H4 and COX-2 into consideration.

As a novel member of IQGAPs family, a host of researches were conducted to explore the role of IQGAP3 in cancers. IQGAP3 was previously proved to be involved in cell proliferation, adhesion, migration and metastasis in different cancers [27–29]. Yang et al. revealed the involvement of IQGAP3 in lung cancer development and the molecular mechanisms in tumorigenesis [30]. Shi et al. found that IQGAP3 was a regulator of epithelial-mesenchymal transition by activating TGF-β signaling pathway and a high IQGAP3 level was associated with poor survival of hepatocellular carcinoma patients [31]. Qian et al. indicated that IQGAP3 is a novel biomarker in the diagnosis of hepatocellular carcinoma by detecting plasma IQGAP3 levels [32]. B7-H4 is a ligand of B7 family which has been indicated as a negative regulator of T cell-mediated immunity [33]. Besides, B7-H4 over-expression has been found in lung cancer, ovarian cancer and breast cancer [34–36]. Higher serum B7-H4 was reported to be correlated with diagnosis and prognostic prediction in CRC patients [11]. COX-2 has been proven to play an important role in tumors development and correlated with higher stage tumors, including CRC [19].

Carcinogenesis and development of CRC are multistep which is resulted from cumulative effects of many genes [37]. Besides, considering there has been no study on evaluating the diagnostic or prognostic value of IQGAP3 for CRC. In this study, we detected the serum and tissue IQGAP3 levels and made a comparison with previously reported B7-H4 and COX-2 to assess its diagnostic and prognostic value for CRC for the first time.

We found that serum and tissue IQGAP3, B7-H4 and COX-2 expression levels were all correlated with T stage, N stage and TNM stage. Wang et al. reported that B7-H4 overexpression was correlated with tumor size, lymph node metastasis and tumor infiltration [11]. However, no relationship was found in tumor size in our study. Positive B7-H4 expression was related with tumor infiltration depth and lymph node metastasis [18]. These may be because B7-H4 can provide negative signals to limit T-cell immune response [33]. Albasri et al. also found that high COX-2 expression was associated with high tumor stage [19], which was corresponded with our results. This may be because COX-2 can (1) increase the production of prostaglandins and inhibit the immune response, (2) promote tumor angiogenesis, (3) promote cell proliferation and inhibit cell apoptosis [38]. IQGAP3 was reported to interact with Erk1 and to enhance Erk1 phosphorylation after treatment with epidermal growth factor (EGF) [30]. For Erk signaling promotes cell proliferation [39], it is likely to play a significant role in promoting CRC cell proliferation through interaction with IQGAP3.

When comparing the ROC curves of the three tumor markers, IQGAP3 appeared to be the best in its predictive efficacy. However, when we combined IQGAP3 with B7-H4 or COX-2, we were pleasantly surprised to discover that the IQGAP3 + COX-2 AUC was much higher than that of other two-combined markers and single markers. When we combined the three markers together, IQGAP3 + B7-H4 + COX-2 showed the best diagnostic power in this study. Wang et al. revealed that the combination detection of B7-H4 and CEA increased the sensitivity and specificity in CRC diagnosing compared to single marker detection [11]. Yang et al. found that serum COX-2, CEA and CA199 were highly expressed in CRC and can be used as indicators for the early diagnosis of CRC [20]. IQGAP3 was previously proved to have a diagnostic value and function as a regulator of metastasis in hepatocellular carcinoma [31, 32]. Similarly, we found its diagnostic value in CRC. Although B7-H4 and COX-2 were previous shown to be of diagnostic value in CRC patients, we really found the diagnostic value of IQGAP3 was better than these two markers, with a highest sensitivity and AUC of the three. However, the specificity of IQGAP3 was the lowest, which resulted in false positive rate being too high. To avoid this deficiency, we think it is better to combine IQGAP3 with B7-H4 or COX-2 when detecting CRC.

According to our survival analysis, we found that higher expression of IQGAP3, B7-H4 and COX-2 in serum or tissue all indicated poor survival. Similar results were found that COX-2 play a significant role in carcinogenesis and progression of CRC [19], which may be because COX-2 can damage the immune system and promote tumor invasion [38]. CRC patients with high expression of B7-H4 had poorer survival than those with low B7-H4 expression [33]. As a member of B7 family, B7-H4 may provide negative signals to inhibit or reduce immune response [18]. So far no studies have been found demonstrating the relationship between IQGAP3 and CRC, our findings may verify the role of IQGAP3 played in tumorigenesis and tumor progression [40], and can further clarify the prognostic value of IQGAP3 in CRC patients.

There are still some limitations in this study. We only included adenocarcinoma patients from a single center and the sample size was not large enough, which may result in selection bias. A multicenter study with a larger sample size is warranted in the future to avoid inflating the sensitivity and specificity of the biomarkers. Besides, in this study we only demonstrated that IQGAP3 has a prognostic value, a further prospective study is warranted to explore which is the best predictor among IQGAP3, B7-H4 and COX-2 in CRC patients. Lastly, the regulation mechanism of IQGAP3 in the CRC progression need further exploration.

## Conclusions

In summary, as a novel tumor marker, IQGAP3 has a better diagnostic efficacy than B7-H4 and COX-2 in detecting CRC. Combining IQGAP3 with B7-H4 or COX-2 presents a more excellent result. Moreover, similar to B7-H4 and COX-2, IQGAP3 is of value in prognostic evaluation in CRC patients.

## Additional file

**Additional file 1.** Correlation of serum IQGAP3, CEA and CA19-9 levels with clinicopathological features in 118 CRC patients. One hundred eighteen CRC patients who met the inclusion criteria were analyzed in this study. The correlation between clinicopathological characteristics and serum tumor markers were analyzed.

## Data Availability

The datasets used and/or analyzed during the current study are available from the corresponding author on reasonable request.

## Abbreviations

IQGAP: the IQ-motif-containing GTPase-activating protein
COX-2: cyclooxygenase-2
CRC: colorectal cancer
ROC: the receiver operating characteristics curve
AUC: the area under the curve
CEA: carcinoembryonic antigen
EDTA: ethylene diamine tetraacetie acid
OS: overall survival
CV: coefficient of variation
