# Supplementary material for: Clinical value of detecting IQGAP3, B7-H4 and cyclooxygenase-2 in the diagnosis and prognostic evaluation of colorectal cancer
Source: Cancer Cell Int. 2019 Jun 14;19:163. doi: 10.1186/s12935-019-0881-3 (PMC6570966; doi:10.1186/s12935-019-0881-3)
Supplement: Supplementary file 1 — Additional file 1. Correlation of serum IQGAP3, CEA and CA19-9 levels with clinicopathological features in 118 CRC patients. One hundred eighteen CRC patients who met the inclusion criteria were analyzed in this study. The correlation between clinicopathological characteristics and serum tumor markers were analyzed. [file 12935_2019_881_MOESM1_ESM.docx]

| Parameters | Patients | IQGAP3 | *P* | CEA | *P* | CA19-9 | *P* |
| --- | --- | --- | --- | --- | --- | --- | --- |
|  | n (%) | Mean±SD, pg/mL |  | Mean±SD, ng/mL |  | Mean±SD, U/mL |  |
| Age (years) |  |  | 0.023^a^ |  | 0.082^a^ |  | 0.102^a^ |
| ≤ 60 | 53 (44.9) | 300.26±104.31 |  | 5.62±3.40 |  | 29.62±9.71 |  |
| > 60 | 65 (55.1) | 322.47±99.67 |  | 6.70±2.71 |  | 31.31±9.18 |  |
| Sex |  |  | 0.506^a^ |  | 0.073^a^ |  | 0.188^a^ |
| Female | 49 (41.5) | 316.03±104.98 |  | 6.26±3.70 |  | 30.63±9.93 |  |
| Male | 69 (58.5) | 311.54±101.42 |  | 6.19±3.66 |  | 30.89±9.22 |  |
| Tumor Site |  |  | 0.208^a^ |  | 0.196^a^ |  | 0.234^a^ |
| Colon | 66 (55.9) | 316.38±99.03 |  | 6.18±4.31 |  | 30.12±10.06 |  |
| Rectum | 52 (44.1) | 312.35±103.54 |  | 6.28±4.76 |  | 31.12±8.59 |  |
| Tumor Size (cm) |  |  | 0.052^a^ |  | 0.358^a^ |  | 0.162^a^ |
| ≤ 4 | 63 (53.4) | 311.93±90.17 |  | 6.28±4.93 |  | 30.28±9.11 |  |
| > 4 | 55 (46.6) | 325.00±87.65 |  | 6.16±3.97 |  | 30.90±9.83 |  |
| T Stage |  |  | 0.001^b^ |  | 0.024^b^ |  | 0.016^b^ |
| T1 | 22 (18.6) | 180.33±71.10 |  | 5.34±1.17 |  | 27.19±8.87 |  |
| T2 | 32 (27.1) | 185.57±84.10 |  | 5.92±1.85 |  | 29.81±9.43 |  |
| T3 | 34 (28.8) | 272.78±79.20 |  | 6.08±3.82 |  | 30.90±10.16 |  |
| T4 | 30 (25.4) | 382.71±76.76 |  | 6.34±2.14 |  | 31.5±9.58 |  |
| N Stage |  |  | <0.001^b^ |  | 0.004^b^ |  | 0.008^b^ |
| N0 | 48 (40.7) | 252.97±88.16 |  | 5.8±1.79 |  | 27.71±9.24 |  |
| N1 | 39 (33.1) | 323.11±80.03 |  | 6.36±2.67 |  | 30.19±8.94 |  |
| N2 | 31 (26.3) | 392.02±88.68 |  | 6.80±3.43 |  | 32.19±10.87 |  |
| Differentiation degree |  |  | 0.086^b^ |  | 0.022^b^ |  | 0.009^b^ |
| Well | 56 (47.5) | 272.75±75.60 |  | 5.69±1.28 |  | 27.34±9.59 |  |
| Moderate | 50 (42.4) | 351.91±90.22 |  | 6.08±2.55 |  | 30.78±7.65 |  |
| Poor | 12 (10.2) | 358.57±91.57 |  | 6.65±3.64 |  | 31.74±9.03 |  |
| Retrieved LN |  |  | 0.017^a^ |  | 0.085^a^ |  | 0.016^a^ |
| ≤ 12 | 61 (51.7) | 354.78±88.75 |  | 6.32±4.22 |  | 31.01±9.74 |  |
| > 12 | 57 (48.3) | 266.63±96.23 |  | 6.29±3.99 |  | 29.93±10.02 |  |
| TNM Stage |  |  | <0.001^a^ |  | 0.002^a^ |  | <0.001^a^ |
| Ⅰ+Ⅱ | 48 (40.7) | 270.22±102.70 |  | 5.83±3.82 |  | 28.96±8.83 |  |
| Ⅲ | 70 (59.3) | 338.86±92.78 |  | 6.62±2.82 |  | 31.94±9.95 |  |

LN, lymph nodes; TNM, tumor-node-metastasis; SD, standard deviation

^a^By Mann-Whitney U test

^b^By Kruskal-Wallis test

Additional file 1. Correlation of serum IQGAP3, CEA and CA19-9 levels with clinicopathological features in CRC
